# Reduced Dynamic Coupling Between Spontaneous BOLD-CBF Fluctuations in Older Adults: A Dual-Echo pCASL Study

**DOI:** 10.3389/fnagi.2018.00115

**Published:** 2018-04-23

**Authors:** Piero Chiacchiaretta, Francesco Cerritelli, Giovanna Bubbico, Mauro Gianni Perrucci, Antonio Ferretti

**Affiliations:** ^1^Department of Neuroscience, Imaging and Clinical Sciences, Università degli Studi G. d’Annunzio Chieti e Pescara, Chieti, Italy; ^2^Institute for Advanced Biomedical Technologies (ITAB), Università degli Studi G. d’Annunzio Chieti e Pescara, Chieti, Italy; ^3^Clinical-Based Human Research Department—C.O.M.E. Collaboration ONLUS, Pescara, Italy

**Keywords:** aging, arterial spin labeling, BOLD-CBF coupling, fMRI, resting-state

## Abstract

Measurement of the dynamic coupling between spontaneous Blood Oxygenation Level Dependent (BOLD) and cerebral blood flow (CBF) fluctuations has been recently proposed as a method to probe resting-state brain physiology. Here we investigated how the dynamic BOLD-CBF coupling during resting-state is affected by aging. Fifteen young subjects and 17 healthy elderlies were studied using a dual-echo pCASL sequence. We found that the dynamic BOLD-CBF coupling was markedly reduced in elderlies, in particular in the left supramarginal gyrus, an area known to be involved in verbal working memory and episodic memory. Moreover, correcting for temporal shift between BOLD and CBF timecourses resulted in an increased correlation of the two signals for both groups, but with a larger increase for elderlies. However, even after temporal shift correction, a significantly decreased correlation was still observed for elderlies in the left supramarginal gyrus, indicating that the age-related dynamic BOLD-CBF uncoupling in this region is more pronounced and can be only partially explained with a simple time-shift between the two signals. Interestingly, these results were observed in a group of elderlies with normal cognitive functions, suggesting that the study of dynamic BOLD-CBF coupling during resting-state is a promising technique, potentially able to provide early biomarkers of functional changes in the aging brain.

## Introduction

Since the first observation that spontaneous Blood Oxygenation Level Dependent (BOLD) signal fluctuations in the left and right motor cortex are correlated in the absence of a task (Biswal et al., 1995), resting-state fMRI has witnessed an exponential growth of interest.

Since it does not require any task, resting-state fMRI is particularly attractive for studies on patients, children and elderlies, reducing problems related to participant’s compliance or intersubject variability due to task performance. Indeed, there is an increasing number of investigations using resting-state fMRI as a sensitive biomarker to study normal and pathological aging (D’Esposito et al., 1999; Fox and Greicius, 2010; Brier et al., 2014; Gardini et al., 2014; Li et al., 2015; Vecchio et al., 2015; Esposito et al., 2018).

Most fMRI studies are based on the BOLD technique (Bandettini et al., 1992; Kwong et al., 1992; Ogawa et al., 1992) which offers a large sensitivity and easy of implementation. However, due to the complex nature of the BOLD effect, the quantitative interpretation of this fMRI signal can be problematic. Indeed, the BOLD signal change is modulated by local variations in deoxyhemoglobin content stemming from changes in cerebral blood flow (CBF), cerebral blood volume (CBV) and cerebral metabolic rate of oxygen consumption (CMRO_2_) induced by neuronal activation or vascular challenges (Buxton, 2012). Depending on the complex interplay of these variables, the magnitude and dynamics of the BOLD signal change may not always reflect the underlying change in neuronal activity or the variation of a specific hemodynamic/metabolic quantity (Ances et al., 2008; Buxton, 2010; Griffeth and Buxton, 2011; Moradi et al., 2012). This limitation is especially important in studies comparing populations with different neurovascular properties (Liu, 2013). In particular, this issue has been largely recognized when comparing elderly vs. young adults since significant vascular changes are known to occur during adult life (O’Rourke and Hashimoto, 2007; Samanez-Larkin and D’Esposito, 2008; Chen et al., 2011; Lu et al., 2011; Gauthier and Hoge, 2013; De Vis et al., 2015).

To overcome the problem of BOLD signal ambiguity, the acquisition of concurrent BOLD and CBF data from arterial spin labeling (ASL) sequences has been proposed in an early biophysical model that allows the calculation of fractional changes of the different hemodynamic and metabolic variables involved in brain activation (Davis et al., 1998). ASL uses magnetically labeled arterial blood water as an endogenous tracer (Detre et al., 1992) and can quantify both baseline levels and activity induced variation of regional CBF. The Davis model is currently applied with different strategies, usually requiring additional calibration measurements based on gas challenges (Davis et al., 1998; Hoge et al., 1999; Chiarelli et al., 2007) and has been expanded to allow the measurement of absolute CMRO_2_ changes or baseline levels of oxygen consumption (Bulte et al., 2012; Gauthier et al., 2012; Wise et al., 2013; Germuska and Bulte, 2014; Germuska et al., 2016). Although gas challenges might be less tolerated by patients or elderlies, applications of these techniques to aging studies have been recently reported (Mohtasib et al., 2012; Hutchison et al., 2013; Liu et al., 2013; De Vis et al., 2015; Garrett et al., 2017). Alternative calibration techniques without gas administration have also been proposed based on particular MRI acquisition sequences or breath-hold tasks (Kastrup et al., 1999; Fujita et al., 2006; Bulte et al., 2009; Blockley et al., 2012, 2015). Nevertheless, the calibrated BOLD technique has been mostly applied and validated in studies using block paradigms, and its extension to more dynamic experimental designs is not straightforward (Kida et al., 2006; Simon and Buxton, 2015).

In this regard, however, the combined acquisition of BOLD and CBF data using ASL has recently attracted increasing interest to study brain function, even without calibration measurements (Chen et al., 2015; Simon and Buxton, 2015; Storti et al., 2017).

Indeed, although CBF is still an indirect measurement of cerebral metabolism and neuronal activity, it constitutes a well defined and fundamental physiological process that is altered in different pathologies and with physiological aging. Furthermore, despite ASL has lower sensitivity compared to BOLD, it can extend the study of resting-state brain function beyond that of functional connectivity, allowing quantitative CBF measurements and the investigation of BOLD-CBF coupling when the two signals are acquired simultaneously. In particular, the possibility to capture spontaneous fluctuations of cerebral blood flow with ASL has recently received an increasing attention (De Luca et al., 2006; Chuang et al., 2008; Fukunaga et al., 2008; Zou et al., 2009; Viviani et al., 2011; Liang et al., 2014; Tak et al., 2014; Chen et al., 2015; Fernández-Seara et al., 2015; Jann et al., 2015).

This is an appealing application of concurrent BOLD and CBF dynamic data acquisition, potentially able to offer insights on mechanisms underlying resting-state brain functioning and physiology. In this regard, recent evidence demonstrated that the resting-state dynamic relationship between BOLD and CBF is approximately linear across the brain (Fukunaga et al., 2008; Wu et al., 2009), with a significantly stronger coupling between spontaneous BOLD and CBF fluctuations within the major nodes of established resting-state networks (Tak et al., 2014; Cohen et al., 2017). Noteworthy, the study of BOLD-CBF dynamic coupling during resting-state could offer innovative metrics to assess brain health (Chen et al., 2015), in addition to the more commonly used functional connectivity metrics. However, to the best of our knowledge, no investigation based on this method has been performed on aging so far.

In the present study we investigated how the dynamic BOLD-CBF coupling during resting-state is affected by aging. Specifically, we compared a group of healthy elderlies with a group of young subjects, addressing between-group differences in: (i) the linear correlation between the two signals; and (ii) the effect on the calculated correlation when introducing a relative temporal shift between BOLD and CBF timecourses.

## Materials and Methods

Fifteen healthy young adults (Young: mean age = 26.4, SD = 4.2) and 17 healthy elderlies (Elderly: mean age = 63.4, SD = 8.1) were included in the study. All individuals were right-handed, gave their written informed consent according to the Declaration of Helsinki (World Medical Association Declaration of Helsinki, 1997) and all procedures were approved by the Ethics Committee for Biomedical Research of the provinces of Chieti and Pescara and the “G. D’Annunzio” University of Chieti and Pescara. None of the participants reported a history of neurological or psychiatric disease, or used psychopharmacological drugs. Subjects with any drug or alcohol abuse within the previous 6 months were also excluded to avoid confounding effects on the fMRI signal. Other exclusion criteria included implanted metals, pregnancy and abnormal findings in their structural brain MRI. Mild forms of hypertension and hyperlipidemia were accepted. Six older adults used antihypertensive medication.

Elderlies were screened with Mini Mental State Examination (MMSE; Folstein et al., 1975) to evaluate the global cognitive status (reported score range of included subjects: 25.5 ÷ 28.9), Babcock story test to evaluate prose memory (reported score range: 5.2 ÷ 8.8 for immediate recall, 2.5 ÷ 8.6 for delayed recall), and the Frontal Assessment Battery (FAB) to assess global executive functions (reported score range: 14.7 ÷ 18.3). Young group was screened using the Trail Making Test to evaluate sustained visuo-spatial attention (reported score range: 5.5s ÷ 48.8), MMSE (27.7 ÷ 29.3), Babcock story test (7.2 ÷ 8.9 for immediate recall, 5.1 ÷ 8.8 for delayed recall) and FAB (15.1 ÷ 18.9). Statistical between-groups comparisons were performed with parametric or non-parametric tests, depending on normality distribution verified using the Shapiro test. A significant between-group difference was observed for MMSE (*p* = 0.03, Wilcoxon test), whereas Babcock and FAB tests did not reveal significant effects (*p* = 0.19 and *p* = 0.43 respectively, unpaired *t*-tests).

All participants were required to refrain from caffeine, alcohol and nicotine for at least 6 h before the MRI session.

MRI was performed with a 3T Philips Achieva scanner (Philips Medical Systems, Best, Netherlands), using a whole-body radiofrequency coil for signal excitation and an 8-channel phased-array head coil for signal reception.

Subjects were instructed to keep their eyes closed and not to engage in structured thoughts during acquisition.

Resting-state CBF and BOLD data were simultaneously acquired with a dual-echo pseudo-continuous ASL (pCASL) sequence (Dai et al., 2008) with the following parameters: TR/TE1/TE2: 3500/10/28 ms, FOV 230 mm × 230 mm, matrix 64 × 64, voxel size 3.6 mm × 3.6 mm × 5 mm, SENSE factor 2.3, 19 slices acquired in ascending order, 90 dynamics. The label duration was 1650 ms and the postlabel delay was 1000 ms.

Baseline perfusion was measured using a pCASL sequence optimizing the labeling parameters for a reliable quantification of CBF for both young and elderly subjects (Alsop et al., 2015): TR/TE 4269/10 ms, FOV 230 mm × 230 mm, matrix 64 × 64, voxel size 3.6 mm × 3.6 mm × 5 mm, SENSE factor 2.3, 19 slices acquired in ascending order, 60 dynamics. The label duration was 1750 ms and the postlabel delay was 1900 ms. Background suppression pulses at 2110 ms and 3260 ms after start of labeling were used. The labeling plane of the pCASL sequences was positioned 85 mm below the AC-PC line, according to recent guidelines (Aslan et al., 2010; Alsop et al., 2015). An equilibrium magnetization image (M_0_) was also acquired with scan parameters identical to the pCASL sequence (same matrix and readout) but using a long TR (10,000 ms) and without labeling or background suppression pulses.

A high resolution structural volume was finally acquired via a 3D fast field echo T1-weighted sequence with the following parameters: 1 mm isotropic voxel size, TR/TE = 8.1/3.7 ms, flip angle = 8°, 160 sections, SENSE factor = 2.

During fMRI, physiological signals related to respiratory and cardiac cycles were registered using a pneumatic belt strapped around the upper abdomen and a pulse oximeter placed on a finger of the right hand, respectively. Respiratory and cardiac data were both sampled at 100 Hz and stored in a logfile for each run.

Resting state pCASL fMRI data were analyzed using AFNI (Cox et al., 2006^1^) and custom-written software implemented in Python^2^. First, the dual-echo pCASL data were split into four EPI timeseries, corresponding to label and control images for acquisitions at TE1 and TE2.

Then, initial preprocessing was performed for both echoes on the label and control ASL images separately (Restom et al., 2006; Wang et al., 2008) according to the following steps:

(i) RETROICOR was applied to remove signal fluctuations related to cardiac and respiratory cycles (Glover et al., 2000);
(ii) slice-timing correction using sinc interpolation;
(iii) motion correction was performed using rigid body registration to realign all time frames to a base image represented by the first label/control volume (dummy scans prevented the need to discard the first few volumes). A summary statistic of motion was defined as the root mean square (RMS) of the six realignment parameters (three translations and three rotations), in order to inspect for EPI timeseries affected by excessive motion. One elderly subject exceeded an RMS value of 1.5 mm for both label and control images and was discarded from further analysis. Further motion assessment was performed considering the RMS value of the differentiated EPI timeseries (DVARS) within a whole brain spatial mask (Power et al., 2012, 2014). The run-averaged DVARS metrics was then compared across groups using ANOVA in order to characterize potential motion effects not accounted for by spatial registration and regression of motion parameters;
(iv) an additional coregistration was performed to minimize effects of the spatial offset between label and control timeseries caused by possible head motion between the reference control image and the reference label image. The corresponding spatial transformation was determined using the mutual information-based approach (Studholme et al., 1998) which is much less sensitive to the labeling related signal intensity differences that could be interpreted by the standard rigid body motion correction algorithm as apparent head motion between label and control images (Friston et al., 1995; Woods et al., 1998).

After these steps, the coregistration matrix between the structural data set and the preprocessed timeseries was determined using an affine transformation.

Then, additional preprocessing was performed using ANATICOR (Jo et al., 2010). Briefly, we first obtained individual masks of large ventricles and white matter from the structural scans segmentation using FreeSurfer^3^. The white matter mask was slightly eroded (one functional voxel) to prevent partial volume effects that might include signal from gray matter voxels in the mask. This step was not performed for the CSF mask, since with the functional voxel size used in this study the eroded CSF mask would not contain enough voxels in most subjects. Then, for each run, a global nuisance regressor was obtained extracting the EPI average time course within the ventricle mask and local nuisance regressors were obtained calculating for each gray matter voxel the average signal time course for all white matter voxels within a 3 cm radius (Jo et al., 2010). These nuisance regressors and the six regressors derived from motion parameters were removed from the EPI timeseries using AFNI’s @ANATICOR.

At this stage, pCASL time series with interleaved control and label volumes were rebuilt using the preprocessed data. These preprocessed pCASL data were then temporal filtered to separate CBF and BOLD signals (Chuang et al., 2008). Briefly, the ASL time series can be view as the sum of a component with a rapid modulation reflecting the alternating control and label images, and a component that is not modulated by the labeling process, reflecting slower BOLD weighted signal variations. An high-pass filtering with a cut-off frequency corresponding to 1/4TR has been demonstrated to be effective in retaining the modulated component while minimizing the BOLD contamination (Chuang et al., 2008; Tak et al., 2014). Note that this high-pass filtering does not restrict the CBF timeseries to the high frequency band since the CBF signal is derived afterward from the difference between subsequent scans (i.e., control—label). Specifically, CBF timeseries were obtained from TE1 data by high-pass filtering the corresponding preprocessed pCASL signal (>0.071 Hz, corresponding to 1/4TR), multiplying it by cos[π(*n* − 1)] (where *n* is the frame number), and then summing together every two images. BOLD timeseries were obtained from TE2 data by low-pass filtering (<0.071 Hz) the corresponding preprocessed pCASL signal and then summing together every two images.

After these processing procedures, both BOLD and CBF data were spatially normalized using Advanced Normalization Tools (ANTs; Avants et al., 2009). Briefly, the individual T1-weighted anatomical images of young and elderly subjects were first bias corrected using the N4ITK algorithm (Tustison et al., 2010) and then used to create a study specific template encompassing the age range in our study (Avants et al., 2010). Non-brain removal was also performed using BET (Smith, 2002) and a nonlinear warping of the resulting template to the MNI standard space (3 mm isotropic spatial resolution) was computed using symmetric diffeomorphic image normalization (Avants et al., 2008). Finally, BOLD and CBF data were spatial smoothed (6 mm FWHM) and band-pass filtered (0.01–0.071 Hz).

After these preprocessing procedures, linear correlation (Pearson *r*) between BOLD and CBF timeseries was calculated for each voxel to obtain individual maps representing the BOLD-CBF coupling during resting-state. In this step no time-shift was introduced between the two timecourses and the resulting correlation coefficients were indicated as r_0_. Random effects group maps (one sample *t*-test) were then obtained after individual r_0_ to z-Fisher transform for Young and Elderly separately and then compared between groups (two-sample unpaired *t*-test). Group maps were thresholded at *p* < 0.05, FDR corrected.

In a second step, the cross-correlation between BOLD and CBF timecourses was calculated again after introducing a variable time-shift τ in the BOLD signal (eq.1), following previous work (Fukunaga et al., 2008; Tak et al., 2014). In this calculation, both BOLD (t) and CBF (t) were first upsampled to a resolution of 100 ms using sync interpolation as implemented in the AFNI program *3Dtshift*. The maximum correlation value (r_max_) was retained for τ ranging between ±7 s (with a 350 ms step). Then, after transforming individual r_max_ to z-Fisher, the random effects group maps were calculated again for Young and Elderly and compared between groups.

(1) $$r_{\max}\;=\;\max_{\tau }\sum_{t}BOLD(t\;+\;\tau )CBF(t)\;-7s\;<\;\tau \;<\;7s$$

Note that previous studies mostly considered the r_max_ approach, in order to maximize the statistical significance of correlation by correcting for potential temporal mismatch between the two hemodynamic signals (Fukunaga et al., 2008; Tak et al., 2014). Here we investigated both r_0_ and r_max_ and in particular how the two groups compare with respect to these metrics. Our choice was motivated by the fact that the temporal mismatch between BOLD and CBF might be group dependent (due to e.g., age-related vascular effects). A quantitative estimation of the effect of time-shift correction on the correlation coefficient was performed in selected regions of interest (ROIs) as follows.

We focused on ROIs in the default mode network (DMN) and frontoparietal network (FPN) that are the two most investigated brain networks. In order to allow the definition of ROIs independent from the previous analysis, independent component analysis (ICA) was performed, using a probabilistic algorithm (Beckmann and Smith, 2004) as implemented in the MELODIC tool of FSL (FMRIB Software Library). Briefly, individual functional datasets were first temporally concatenated across subjects, groups (Young and Elderly) and modalities (BOLD and CBF) to form a single 4D data set to be used as input for the probabilistic ICA algorithm. The number of components was fixed to 20. Dual-regression (Beckmann et al., 2009) was then used to identify individual spatial maps for each independent component to be used as input for the second-level group analysis. In this calculation random-effects group statistical maps were obtained for each component using permutation-based non-parametric testing (5000 permutations; Nichols and Holmes, 2002). Multiple comparisons correction was addressed applying a cluster-based threshold of *Z* > 2.3 and a family-wise-error (FWE) corrected cluster significance of *p* < 0.01 for the suprathreshold clusters. Using these group maps, the DMN and FPN were easily identified from their characteristic spatial pattern, including posterior cingulate cortex, bilateral angular gyrus and ventromedial prefrontal cortex for DMN, bilateral inferior parietal lobe and bilateral middle frontal gyrus for FPN. For our ROIs definition we pooled BOLD and CBF data because a high degree of spatial overlap has been shown for the two modalities in both resting state and task paradigms (Mayhew et al., 2014; Jann et al., 2015). Furthermore, in order to consider gray matter voxels only, a binary mask was defined by averaging the normalized individual gray matter masks obtained from the FreeSurfer segmentation and thresholding at 0.3 (Jann et al., 2015). The final masks representing the investigated ROIs were defined multiplying this binary mask with the ICA clusters.

Then, mean r_0_ and r_max_ values were extracted from these ROIs and the r_max_ − r_0_ difference was compared between groups (two-sample unpaired *t*-test). Correction for multiple comparisons was performed using FDR. We expect this difference to be larger for elderlies due to an increased time-shift between BOLD and CBF timecourses, possibly reflecting age related modifications of vascular response.

In addition, we computed the resting state fluctuation amplitude (RSFA; Kannurpatti and Biswal, 2008) for both BOLD and CBF timeseries in Elderly and Young, using the AFNI program *3dRSFC*. The corresponding values were extracted from our ROIs and compared between groups (two-sample unpaired *t*-tests with correction for multiple comparisons using FDR). This analysis aimed at evaluating the impact of potential age dependent differences in the amplitude of resting state BOLD and CBF fluctuations on the observed linear correlation between the two signals, since a reduced fluctuation amplitude could lead to a decreased correlation coefficient in presence of noise (Liu, 2013).

Finally, we also extracted regional perfusion values from quantitative CBF maps provided by the background suppressed ASL data and a single compartment model (Buxton et al., 1998; Alsop et al., 2015):

(2) $$CBF\;=\;\frac{6000\lambda (SI_{c}-SI_{L})e^{\frac{PLD}{T_{1A}}}}{2\alpha \alpha_{\text{inv}}T_{1\text{A}}M_{0}(1-\;e^{-\;\frac{\tau }{T1\text{A}}})}\;[\text{ml}/100g/\min]$$

Where λ is the blood-brain partition coefficient (0.9 ml/g), SI_C_ and SI_L_ are the means over time of the control and label images respectively, PLD is the slice-dependent post label delay (1900–2800 ms), T_1A_ is the longitudinal relaxation time of arterial blood (1650 ms at 3T), α is the labeling efficiency (0.85; Alsop et al., 2015), α_inv_ is a correction factor for the background suppression (0.83; van Osch et al., 2009), M_0_ is the equilibrium magnetization signal, and τ is the label duration (1750 ms).

## Results

**Figure 1:**
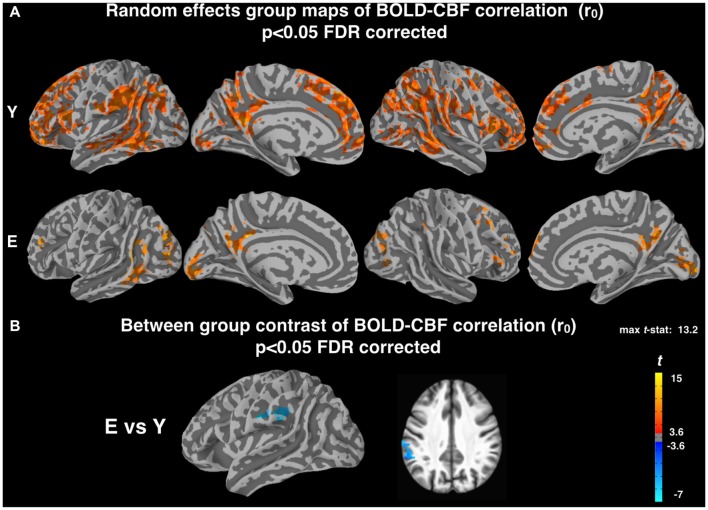
**(A)** Random effect group maps showing the dynamic coupling between spontaneous blood oxygenation level dependent (BOLD) and cerebral blood flow (CBF) fluctuations for Young (Y) and Elderly (E) groups when potential time shifts between the two signals were not compensated for (r_0_, see “Materials and Methods” section). **(B)** Between-group contrast comparing the dynamic coupling between spontaneous BOLD and CBF fluctuations for Y and E, using r_0_ values. A significant age related decrease of BOLD-CBF coupling is observed in the left supramarginal gyrus (MNI coordinates: −59, −34, 33). The statistical maps were thresholded at *p* < 0.05 (corrected for multiple comparisons using FDR) and superimposed on the partially inflated study specific template.

Random effects group maps obtained using the z-Fisher transformed voxelwise correlation (r_0_, see “Materials and Methods” section) between BOLD and CBF timeseries during resting state are shown in Figure 1A for the two groups. In young subjects, a significant correlation between spontaneous fluctuations of BOLD and CBF signals was observed in most cortical areas, whereas in elderly subjects this correlation was markedly reduced (mean r_0_ values in significant areas were 0.24 ± 0.03 for Young and 0.18 ± 0.04 for Elderly). The BOLD-CBF coupling was stronger in medial, parietal and frontal regions, which are part of well-established resting state brain networks. No significant correlation was observed in white matter or other brain structures. The voxelwise contrast between groups showed a significantly lower correlation for elderly subjects in the left supramarginal gyrus (MNI coordinates: −59, −34, 33; Figure 1B).

**Figure 2:**
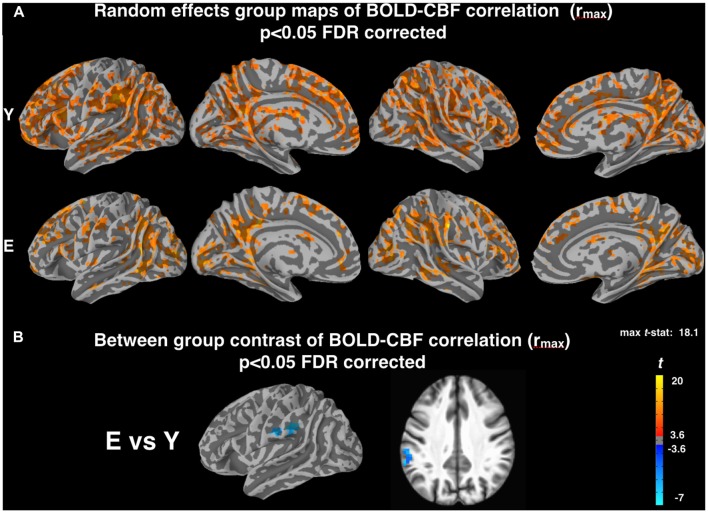
**(A)** Random effect group maps showing the dynamic coupling between spontaneous BOLD and CBF fluctuations for Y and E groups when potential time shifts between the two signals were compensated for (r_max_, see “Materials and Methods” section). **(B)** Between-group contrast comparing the dynamic coupling between spontaneous BOLD and CBF fluctuations for Y and E, using r_max_ values. Note that a significant age related decrease of BOLD-CBF coupling is observed in the left supramarginal gyrus (MNI coordinates: −58, −36, 34) even after temporal shift correction, in a cluster of voxels largely overlapping with that observed in Figure 1B. The statistical maps were thresholded at *p* < 0.05 (corrected for multiple comparisons using FDR) and superimposed on the partially inflated study specific template.

Random effects group maps obtained with the z-Fisher transformed maximum voxelwise correlation between BOLD and CBF timeseries (r_max_, obtained introducing a temporal shift between the two signals as described in “Materials and Methods” section) are reported in Figure 2A. As expected, the number of cortical voxels showing a significant coupling increased for both groups. Interestingly, this increase was more pronounced for Elderly (mean r_max_ values in significant areas were 0.32 ± 0.02 for Young and 0.30 ± 0.03 for Elderly). However, the voxelwise contrast between groups still showed a significantly lower correlation for elderly subjects in the left supramarginal gyrus (MNI coordinates: −58, −36, 34; Figure 2B), in a cluster of voxels largely overlapping with that observed in Figure 1B.

**Figure 3:**
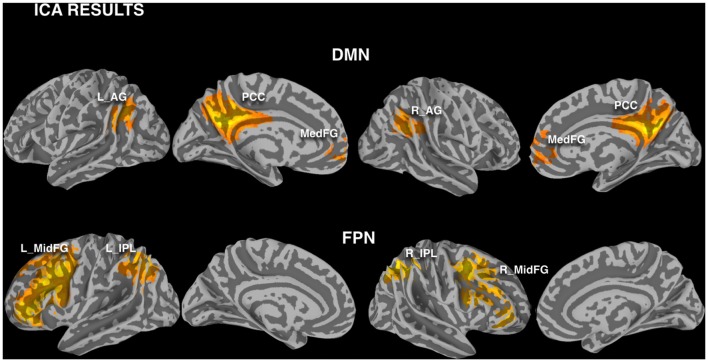
Investigated nodes of default mode network (DMN) and frontoparietal network (FPN), defined with independent component analysis (ICA) calculated pooling the two groups and modalities (FWE corrected cluster significance of *p* < 0.01). Regions of interest (ROIs) were defined masking these clusters with a gray matter binary mask (L_AG/R_AG, left/right angular gyrus; PCC, posterior cingulate cortex; Med_FG, medial frontal gyrus; L_IPL/R_IPL, left/right inferior parietal lobule; L_MidFG/R_MidFG, left/right middle frontal gyrus).

**Figure 4:**
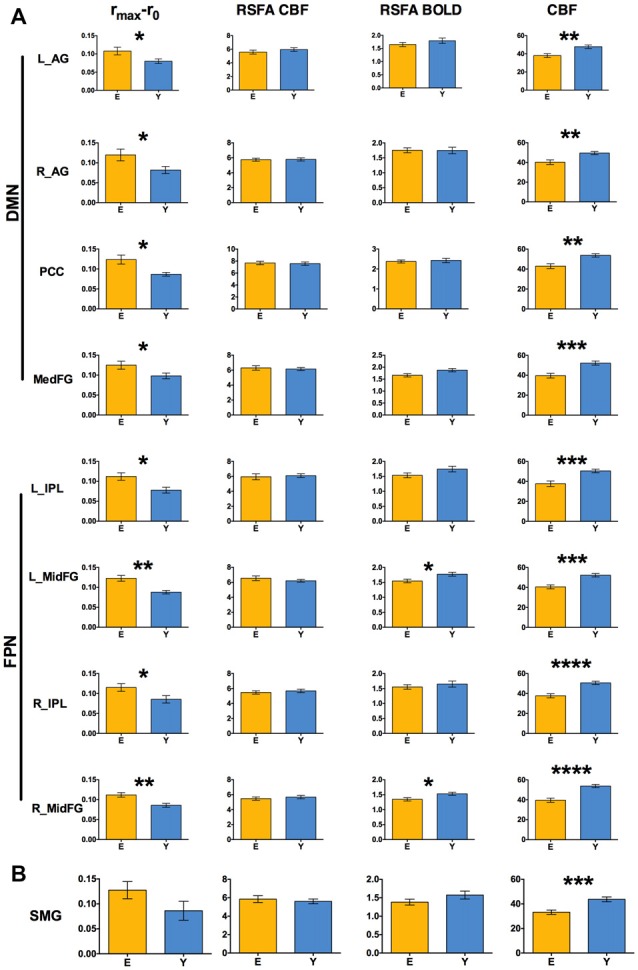
**(A)** Between-group comparison of r_max_ −r_0_ values, resting state fluctuation amplitudes (RSFAs) and baseline perfusion values extracted in ROIs of Figure 3 (two-sample unpaired *t*-test; *p < 0.05 FDR corrected, **p < 0.01 FDR corrected, ***p < 0.001 FDR corrected, ****p < 0.0005 FDR corrected). **(B)** The same values extracted from the ROI obtained pooling the two clusters in Figures 1B, 2B and masking with gray matter. Error bars are standard errors.

DMN and FPN were easily identified in the ICA results, with one component showing the typical DMN pattern, one component showing the left FPN and another component the right FPN (Figure 3). The results of the ROI approach for the selected regions in DMN and FPN are reported in Figure 4A. The comparison of the r_max_ − r_0_ difference between groups showed significantly larger values for Elderly in all ROIs, indicating a more pronounced effect of time-shift corrections on the linear correlation between BOLD and CBF timecourses in elderly subjects. An effect size estimate (Cohen’s *d*) was also computed for the between-group comparison of the r_max_ − r_0_ difference in these ROIs. The observed values were quite large, ranging from 0.78 to 1.24.

A larger r_max_ − r_0_ difference for Elderly with respect to Young was also observed in the supramarginal gyrus, without reaching statistical significance (Figure 4B, values extracted from a mask obtained pooling the clusters in Figures 1B, 2A). The time-shift parameters in the different ROIs ranged from −1.12 s to −1.81 s for Elderly and from −0.4 s to −1.4 s for Young. The between-group difference was significant in the right angular gyrus (−1.8 s for Elderly and −0.5 s for Young, *p* < 0.03, unpaired *t*-test), indicating an increased delay of BOLD dynamics (with respect to CBF) in elderlies.

The baseline CBF values showed the well known age related decrease for all the investigated regions (Figure 3). However, no significant correlation of the BOLD-CBF coupling with baseline CBF values was observed across subjects, for either Elderly or Young groups.

The control analysis on the amplitude of resting state low frequency fluctuations revealed that RSFA values in these regions did not change significantly with age, except for the BOLD-RSFA in the frontal regions of FPN (Figure 4A). However, no region showed a significant across-subjects correlation of the values of BOLD-CBF coupling with BOLD-RSFA or CBF-RSFA values, for either Elderly or Young groups.

The comparison of the run-averaged DVARS metrics did not show statistically significant differences across groups for either control or label images (*F*_(2,120)_ = 1.29; *p* = 0.26). Additional control analysis showed no significant correlation of DVARS values with the BOLD-CBF coupling for either Elderly or Young groups.

## Discussion

The present results showed that in young subjects spontaneous BOLD and CBF fluctuations are significantly synchronized in most cortical areas and especially within the major nodes of prominent resting-state networks, confirming previous evidence (Tak et al., 2014, 2015; Chen et al., 2015). As a new finding, this resting-state BOLD-CBF dynamic coupling was reduced in elderly individuals, especially in the left supramarginal gyrus. Moreover, this decrease was not related to a reduced amplitude of either BOLD or CBF spontaneous fluctuations. Furthermore, elderlies showed a larger increase in the correlation coefficient after the introduction of a relative time-shift between BOLD and CBF timecourses.

Recently, an increasing evidence that intrinsic CBF fluctuations are a major contributor to the resting state BOLD signal has been reported. Indeed, different studies showed a high level of spatial overlap between connectivity maps obtained from BOLD and CBF timecourses (Viviani et al., 2011; Jann et al., 2015). Moreover, a set of studies investigated the dynamic relationship between spontaneous CBF and BOLD fluctuations (Fukunaga et al., 2008; Wu et al., 2009; Tak et al., 2014, 2015; Chen et al., 2015; Cohen et al., 2017). Fukunaga et al. (2008) were the first to investigate the dynamic coupling between BOLD and perfusion fluctuations during resting state, showing that the two signals are correlated in most part of the cortex, with little involvement of white matter and cerebrospinal fluid. Furthermore, using the BOLD/perfusion ratio to target resting state oxidative metabolism fluctuations, they found a similar BOLD/CBF coupling with respect to a visual task induced brain activity, adding evidence to a metabolic/neuronal origin of spontaneous BOLD and CBF fluctuations. In a subsequent work, Wu et al. (2009) obtained functional connectivity maps using CMRO_2_ time series derived from simultaneous BOLD and CBF time series acquired with ASL. Although the CMRO_2_ time courses were estimated assuming the steady-state biophysical BOLD model, that might have some limitations in a more dynamical situation (Simon and Buxton, 2015), the strong similarity observed by Wu et al. (2009) between functional connectivity maps obtained from BOLD, CBF and CMRO_2_ time courses added further evidence to a significant BOLD-CBF coupling during resting state. In a recent work the study of dynamic BOLD-CBF relationship during resting state was addressed with significant methodological improvements, including physiological noise correction in the tag and control ASL images and taking into account the influence of global cardiac fluctuations (Tak et al., 2014). This work demonstrated that the resting-state BOLD-CBF coupling strength, although varying across the brain, was stronger in the gray matter and in particular in the major nodes of well-established functional networks. Moreover, the BOLD-CBF coupling observed by Tak and collaborators was significantly reduced in voxels associated with a high macrovascular content, suggesting that the component of spontaneous BOLD signal fluctuations that is more directly driven by dynamic CBF fluctuations is more likely related to neuronal activity, which is known to modulate the microvascular response.

Keeping in line with previous evidence, our results confirm that in young individuals spontaneous BOLD and CBF fluctuations are significantly synchronized in most cortical areas and especially in regions of the major resting state networks. As a new finding, we observed a general age-related decrease of the BOLD-CBF dynamic coupling during resting state across cortical areas. The between-group voxelwise contrast showed that this decrease was statistically significant in a cluster of voxels in the left supramarginal gyrus.

Slight discrepancies between BOLD and CBF dynamics have been previously reported even in young subjects using e.g., visual or motor stimuli (Obata et al., 2004; Cavusoglu et al., 2012). These discrepancies can be expected, due to possible temporal uncoupling of the involved physiological responses (i.e., CBF, CBV and CMRO_2_) that have a competing effect on the BOLD signal amplitude. Indeed, a temporal uncoupling of e.g., CBF and venous CBV has been proposed in early models trying to explain BOLD signal transients like the post-stimulus undershoot or the initial overshoot during long stimulation blocks (Buxton et al., 1998, 2004; Buxton, 2012). Although a neuronal contribution to these transients has been recognized (Sadaghiani et al., 2009; Mullinger et al., 2014, 2017), there is increasing evidence that vascular mechanisms related to delayed compliance of venous vessels play an important role as well (Chen and Pike, 2009; Havlicek et al., 2017). In particular, a recent work suggests that the venous CBV response can be an order of magnitude slower than either CBF or CMRO_2_ (Simon and Buxton, 2015).

Despite the specific physiological mechanisms responsible for the decreased BOLD-CBF dynamic coupling that we observed in elderly cannot be determined using the present data alone, it could be argued that increased vessel stiffening due to age (Podlutsky et al., 2010; Trott et al., 2011; Csiszár et al., 2015; Tsvetanov et al., 2015; Chiarelli et al., 2017; Tan et al., 2017; Tarantini et al., 2017; Toth et al., 2017) can lead to increased delay in venous compliance, thus introducing further temporal discrepancies between BOLD and CBF timecourses. Animal studies also showed that modifications of vessel compliance with age decrease the ratio of CBV to CBF responses (Dubeau et al., 2011; Desjardins et al., 2014) that in turn would also affect the BOLD response. Moreover, the ratio of CBV to CBF response is also reflected in the Grubb’s parameter (Grubb et al., 1974) that is involved in calibrated BOLD models. Importantly, this parameter has been shown to vary not only with aging (Dubeau et al., 2011) but also during different phases of functional stimulation, leading to transient relationships between CBF, CBV and BOLD changes (Kida et al., 2006), suggesting a possible effect on BOLD-CBF coupling. The potential role of delayed vessel compliance is also supported by our second finding, i.e., that introducing a time-shift correction between BOLD and CBF signals has more effect for elderlies than for young subjects in increasing the calculated correlation coefficient. However, even with time-shift correction, the left supramarginal gyrus showed a significant between-group difference in the voxelwise contrast, indicating that the dynamic uncoupling of BOLD and CBF timecourses in this region is more affected by the aging process and can be only partially explained with a simple time-shift between the two signals. Interestingly, different neuroimaging and transcranial magnetic stimulation studies reported that the left supramarginal gyrus is involved in verbal working memory and episodic memory (Romero et al., 2006; Koelsch et al., 2009; Deschamps et al., 2014; Thakral et al., 2017), which are functions known to be impaired with age. Considering that our investigated population was selected among healthy elderlies that did not show a compromised performance in memory tests, our results acquire an increased interest. Indeed, the observed age related differences in the dynamic BOLD-CBF coupling could be considered a potential biomarker that might anticipate changes in neuronal function. In this regard, this type of index could be spatially more specific than e.g., age-related decrease of baseline perfusion that we and others (Parkes et al., 2004; Ambarki et al., 2015; De Vis et al., 2015) clearly observed in most cortical areas. However, we are aware that a full validation of this hypothesis would require additional data, with a larger sample size and possibly longitudinal studies.

### Potential Limitations and Caveats

As in all studies using dynamic ASL to assess brain function, a major concern is minimizing BOLD contamination of the CBF signal. We addressed this issue by using the short echo time data (which have minimal sensitivity to the BOLD effect) and by high pass filtering the ASL signal followed by demodulation to derive the CBF timecourse (Chuang et al., 2008). This approach can be considered a generalization of previously proposed methods like e.g., sinc interpolation (Liu and Wong, 2005), with similar efficiency in removing BOLD contamination from the perfusion timeseries. Another important concern in resting state fMRI studies is the physiological noise due to cardiac and respiratory activity. While different methods of noise cleanup have been established for the BOLD signal (for a review see; Murphy et al., 2013; Liu, 2016), there is scarce evidence on the application of similar procedures to ASL. We chose the approach using RETROICOR to correct time-locked effects of cardiac and respiratory fluctuations on the EPI signal of label and control separately (Restom et al., 2006). Indeed, modeling the effect of the cardiac and respiratory cycles on the derived CBF weighted timeseries had minimal effect on signal quality (Restom et al., 2006). However, as observed by the same authors, future approaches using additional information such as end tidal carbon dioxide measurements could allow improved modeling of the effect of physiological noise on the perfusion timecourses, extending previous work on BOLD (Wise et al., 2004). An additional source of noise in the fMRI signals is represented by head motion, that has been shown to introduce spurious correlations in functional networks investigated in resting state BOLD studies (Power et al., 2012). This issue is particularly important when comparing different populations that could differ in the amount of head motion, like e.g healthy subjects vs. patients or young subjects vs. elderlies and censoring procedures have been proposed, in addition to regression of motion parameters, to mitigate these effects in resting state BOLD timeseries (Power et al., 2014). Again, the extension of these methods to CBF timeseries is not straightforward because the perfusion information is carried by the difference image between label and control that should be censored in pairs, thus strongly reducing the number of usable timepoints. However, motion parameters derived during preprocessing of label and control images did not differ significantly between groups in our data, suggesting a negligible effect of motion on the observed results.

In addition to noise concerns, potential between-group differences in BOLD and/or CBF signal fluctuation amplitude could also affect the calculated correlations. Indeed, while from a mathematical point of view the linear correlation of two timecourses is not affected by the amplitude of the signals, in presence of noise a reduced fluctuation amplitude could lead to a decreased correlation coefficient (Liu, 2013). However, the two groups showed comparable levels of signal fluctuations for both BOLD and CBF in the majority of investigated ROIs, suggesting no bias related to this issue. Even in the two regions showing a significant decrease of BOLD fluctuations in elderlies, any significant correlation of RSFA with BOLD-CBF correlation was observed. A reduced fluctuation amplitude in elderlies could have been expected due to gray matter atrophy. In this regard, the observation of similar fluctuation amplitudes of the functional signals in the two groups also helps to mitigate the concern of potential confounds due to the presence of atrophy in elderlies that could have biased our results.

Potential biases in the results could also be introduced by the six older adults using antihypertensive medication that would possibly alter the hemodynamic response in these subjects. In our group of subjects a control analysis revealed similar BOLD-CBF coupling when comparing this hypertensive subgroup with the other elderlies, with no significant differences. Nevertheless, future studies should address this issue using larger sample sizes.

A further concern could be raised regarding the postlabel delay (1000 ms for the first slice) used for the study of dynamic BOLD-CBF coupling that can be too short to ensure a complete delivery of the bolus to the tissue at the time of acquisition, in particular for elderlies. However, while this is an important issue for absolute CBF quantification, short postlabel delays are often used or even recommended for functional applications of ASL because offer a larger sensitivity and temporal resolution without introducing bright spots in activation maps due to large vessel contribution or introducing distortions of timecourses (Gonzalez-At et al., 2000; Zappe et al., 2008). In contrast, for the quantification of baseline CBF, we used a longer postlabel delay (1900 ms for the first slice), following current guidelines for both young and healthy elderlies (Alsop et al., 2015).

Finally, another limitation of the study could arise from the small sample size, potentially resulting in a low statistical power. Indeed, despite statistical significance was obtained for the between-group comparison of our main metrics of interest, future studies with larger sample size possibly including elderlies with impaired cognitive functions would allow further assessment of the significance of our findings.

## Conclusion

In this study we observed an age-related decrease of the temporal correlation between the BOLD and CBF spontaneous fluctuations in the cortex. Interestingly, when compensating for potential time-shifts between the two signals, the increase of the correlation coefficient was larger for elderlies. However a significant between-group difference was observed in the left supramargynal gyrus even after time-shift correction, suggesting a more pronounced age-related BOLD-CBF uncoupling in this area. These results suggest that the study of dynamic coupling between spontaneous BOLD-CBF fluctuations using the simultaneous acquisition of the two signals with ASL is a promising technique to study-resting state brain function in aging and disease.

## Author Contributions

PC and AF: conceived and designed the study and wrote the article. FC and GB: enrolled and tested the subjects. PC and MGP: performed the experiments. PC: implemented AFNI and Python scripts. PC, FC and AF: performed the analyses.

## Conflict of Interest Statement

The authors declare that the research was conducted in the absence of any commercial or financial relationships that could be construed as a potential conflict of interest.

## Footnotes

^1^afni.nimh.nih.gov/afni

^2^http://www.python.org

^3^http://surfer.nmr.mgh.harvard.edu
